# Primary extra-uterine and extra-ovarian mullerian adenosarcoma: case report and literature review

**DOI:** 10.1186/s12885-018-4037-y

**Published:** 2018-02-05

**Authors:** Vincenzo Dario Mandato, Federica Torricelli, Valentina Mastrofilippo, Riccardo Valli, Lorenzo Aguzzoli, Giovanni Battista La Sala

**Affiliations:** 1 Unit of Obstetrics and Gynecology, IRCCS- Azienda Unità Sanitaria Locale, Viale Risorgimento n 80, Reggio Emilia, Italy; 2 Laboratory of Translational Research, Azienda Unità Sanitaria Locale, IRCCS, Reggio Emilia, Italy; 3 Unit of Surgical Gynecol Oncology, Azienda Unità Sanitaria Locale, IRCCS, Reggio Emilia, Italy; 4 Unit of Pathology, Azienda Unità Sanitaria Locale, IRCCS, Reggio Emilia, Italy; 5 Unit of Obstetrics and Gynecology, University of Modena e Reggio Emilia, Reggio Emilia, Italy

**Keywords:** Mullerian extra-uterine adenosarcoma, Mullerian extra-genital adenosarcoma, Survival, Vaginal adenosarcoma, Symptoms, Treatment, Review

## Abstract

**Background:**

Extra-uterine mullerian adenosarcomas have varying biological behaviours depending on the presence of endometriosis or sarcomatous overgrowth. These behaviours manifest according to the tumours’ histological characteristics and sites of origin. The best treatment and oncologic outcome have not been clarified because only a few cases of extra-uterine and extra-ovarian adenosarcoma have been described in the literature. Here, we report a case of primary peritoneal adenosarcoma with sarcomatous overgrowth and review all reported cases of adenosarcomas arising outside of the uterus and outside the ovaries to identify the best treatment options and clarify outcomes.

**Case presentation:**

A 79-year-old woman was referred to our Department with an abdominal mass resembling a fibroid with a haemorrhage. Her gynaecological history was negative. A transvaginal and transabdominal ultrasound examination revealed a multicystic mass resembling an ovarian tumour arising from the pelvis and extending up to the abdomen. At laparotomy a peritoneal mass arising from Douglas peritoneum was resected. The uterus and adnexa appeared normal, and a supra-cervical hysterectomy with bilateral salpingo-oophorectomy was performed. No macroscopic residual disease was present. Final pathology diagnosed a malignant peripheral nerve sheath tumors with divergent differentiation. Four weeks later a new, multicystic mass was found. Due to the progressive poor condition, the patient died four months after diagnosis. Histological slides were reviewed by external expert pathologists and the final diagnosis was of extra-genital adenosarcoma with sarcomatous overgrowth. Furthermore, we also collected and analysed articles written in English regarding extra-uterine and extra-ovarian adenosarcomas published between January 1974 and October 2016. PubMed was used as a database for this search. Clinical and pathological characteristics, treatments and outcomes were assessed.

**Conclusions:**

Only 41 cases has been reported in literature. Previous endometriosis and sarcomatous overgrowth showed an inverse effect on prognosis. Endometriosis was confirmed to have a positive effect on disease free survival Complete surgical resection is the mainstay of treatment. A worldwide registry is urgently required to collect data to standardize treatment and to obtain reliable data on prognosis.

## Background

Mullerian adenosarcoma (AS) is a rare mesenchymal and epithelial neoplasm of low malignant potential typically occurring in the uterine corpus in perimenopausal or postmenopausal women [1]. It is a mixed tumour that usually arises as a solitary lesion with a benign but sometimes atypical glandular epithelium and low-grade sarcoma, usually of the endometrial stromal type [2].

The first case of AS was described in early 1974 by Clement and Scully [3]. AS typically arises from the corpus uterus, rarely from the cervix or ovary, and extremely rarely from the vagina or from extra-genital sites such as the peritoneum, retroperitoneum, bladder, liver or colon (Table 1) [4–39].

**Table 1:** Clinical features of 41 patients with extra-genital mullerian adenosarcoma reported in the English literature and of the index case

Reference number	Author	Age (years)	Site	Size (cm)	Tumour markers	Signs and Symptoms at presentation	Treatment	Sarcomatous overgrowth	Endometriosis	Hormonal therapy	Follow-up
4	Douglas	18 gravida	Retroperitoneum	5 × 4.5 × 4	NR	Anorexia, suprapubic-low back pain, loss of weight, vaginal bleeding, preterm delivery. 24 weeks	CHT (MTX)	NR	No	No	DOD 10 weeks later with distant metastasis
5a	Clement	45	Right Pelvic peritoneum (pelvic mass that extended into the rectum and the bladder)	7x7x5	NR	Right lower leg thrombophlebitis, right paravaginal mass	Surgery (partial tumour resection) + RT	NR	No	No	DOD 9 months later due to pelvic recurrence and visceral metastasis
5b	Clement	73	Midline Pelvic peritoneum, displcing the bladder anteriorly	14	NR	Large pelvic mass with bilateral hydronephrosis. Inability to void	Surgery (complete tumour resection)	NR	No	No	DOD 2 months later (postoperatively massive gastric bleeding necessitating a subtotal gastrectomy occurred but after that the patient’s conditions deteriorated gradually-autopsy not done-)
5c	Clement	58	Left Pelvic peritoneum	16x15x8	NR	Large pelvic mass. Urinary urgency, rectal pressure	Surgery (partial tumour resection)	NR	Yes	No	AWD local recurrence 15 months later (RT), lung metastases 45 months later (resected)
6	Bard	46	Right pelvic peritonum (the mass was adherent to the right bladder wall and surrounded the right ureter)	10 × 8	NR	Weakness and pelvic pain in the right lower extremity. Urinary incontinency	Biopsy + RT	NR	NR	No	DOD 11 weeks later with distant sepsis and metastasis
7	Kao	42	Left round ligament	10	NR	NR	Surgery (partial tumour resection) + CHT (Cyt) + RT	NR	No	No	DOD after 10 months due to the tumor
8	Russell	29	Left Broad ligament	7x6x5	N.R.	Lower abdominal pain for 2 months and occasional dyspareunia	Surgery (tumour resection)	NR	No	No	Recurrence after 5 months treated with surgery (hysterectomy, bilateral salpingo-oophorectomy) + RT; Died of melanoma after 9 years.
9	Kerner	32 gravida	Broad ligament	15x10x6 and 10x7x4	NR	Abdominal pain at 28 weeks	Surgery (tumour resection)	No	No	No	AWD omentum and infundibulopelvic ligament recurrence 22 months later
10	Vara	62	Bladder	–	NR	Haematuria, weight loss, suprapubic pain	Surgery (radical cystectomy + urethrectomy)	NR	Yes	No	FOD 12 months
11	Roman	55	Retroperitoneal	NR	NR	NR	Surgery (tumour resection) + RT	NR	Yes	No	First abdominal recurrence 3 years later (resection by thoracoabdominal approach + MPA), a 5 cm perihepatic recurrence 5 years later was completely resected, second perihepatic recurrence 7 years from original tumour (resection of recurrence and TMX), 6 months later intrahepatic metastasis (CHT for recurrences (cisplatin-ifosfamide, ifosfamide, doxorubicin), later atrial tumour (resection of cardiac tumour and oral therapy with etoposide). Died after 10 years from original tumour and 70 days after resection of cardiac tumour.
12	De Jonge	16	Pelvic peritoneum and infracolic omentum	NR	Ca 125 > 190 U/ml	Severe Abdominal distension and pain	Surgery (tumour resection with extirpation of pelvic mass, left fallopian tube, infracolic omentum and appendix)	Yes	No	NR	Three weeks after primary surgery a pelvic mass recurred (CHT with doxorubicin and ifosfamide), first recurrence of the pouch of Douglas 8 months later (CHT cisplatinum, etoposide and ifosfamide), second recurrence of the pouch of Douglas 14 months later the first recurrence (bilateral salpingo-oophorectomy, abdominal hysterectomy, pelvic and para-aortic lymphadenectomy and hormonal therapy) FOD 57 months after the last cycle of chemotherapy
13	Benda	65	Vaginal apex	10x7x8	NR	Pelvic pressure and urinary frequency	Surgery (tumour resection)	NR	No	No	Three years later a 6 cm vaginal recurrence was completely resected; 5 yrs. after first recurrence a 12 cm vaginal recurrence was completely resected and a progesterone therapy was delivered; 7 years after the second recurrence a 17 cm pelvic recurrence was partially resected and a TMX (2 weeks) therapy followed by P (2 weeks) therapy was delivered; 10 months after the third recurrence a fourth pelvic recurrence was partially resected and treated with RT. AWD 16 years
14	Ostor	49	Pouch of Douglas	19x8x3	NR	Right Iliac fossa pain	Surgery (tumour resection and abdominal hysterectomy and bilateral salpingo-oophorectomy) Radiotherapy and Hormonal therapy (Medroxyprogesterone)	NR	No	No	AWD recurrence 5 weeks later (chemotherapy cisplatin and ifosfamide). Persistence of some nodularity on the pelvic floor 18 months later
15	Inoue	54	Left paracolpium	15x11x10	Ca 125: 860 U/ml	Brownish vaginal discharge Small ulcer in the left posterior fornix	RT+ Surgery (tumour resection+ total abdominal hysterectomy, bilateral adnexectomy, abdominal perineal resection with colostomy)	NR	Yes	No	FOD 1 year later
16	Judson	42	Vaginal cuff	6 × 3	NR	Endometriosis recurred three times and was treated with surgery, hormonal therapy (megestrol, danazol) and brachytherapy. Lesion coming out from vagina	CHT (paclitaxel and carboplatin followed by TMX)	NR	Yes	No	12 months later a 4 cm vagina recurrence was excised and a RT was delivered than was FOD
17	N’Senda	54	Liver	20 × 12	CA 15–3 and CA 19–9, were three and fourfold normal level respectively	Right-sided epigastric pain	Surgery (tumour resection: A segment -IV enlarged right hepatectomy extended to adjacent diaphragm)	No	Yes	HRT	FOD 24 months
18	Kato	20	Abdominopelvic peritoneum	23x23x14	CA 125: 1000 IU/ml	Fatigue and constipation	Surgery (tumour resection)	NR	No	No	FOD 1 years later
19a	Yantiss	36	Sigmoid	10,5	NR	Hypermenorrhoea, 6 months abdominal pain	Surgery (tumour and sigmoid resection)	NR	Yes	NR	FOD 36 months
19b	Yantiss 2000	50	Colon	NR	NR	NR	Surgery (tumour and colon resection)	NR	Yes	NR	FOD 24 months
19c	Yantiss 2000	83	Small bowel	15	NR	Abdominal mass obstruction	Surgery (tumour and bowel resection)	NR	Yes	NR	NR
19d	Yantiss 2000	43	Small bowel	6.5	NR	NR	Surgery (incomplete tumour resection)	NR	NR	ER T	NR
20	Visvalingam	50	Abdominopelvic peritoneum	13 kg	NV	Painless abdominal swelling	Surgery (tumour resection), Hormonal therapy (progesterone)	NR	No	No	Ten months later a 50 cm pelvic recurrence was resected (tumour debulking, extrafascial hysterectomy, omentectomy, appendicectomy). DOD 16 months later (Autopsy revealed tumor nodules throughout the abdominal and pelvic cavity limited to the peritoneal surface)
21	Anderon	46	Vagina	10 cm	NR		Removal of the vaginal mass, stalk and paravaginal tissue.	No	Yes	Yes	FOD after a parametrium recurrence that was treated with external radiotherapy and interstitial brachytherapy.
22	Dincer	50	Perisplenic Peritoneum	NR	NR	Large bowel obstruction in a woman with Endometriosis treated with aromatase inhibitor	Surgery (partial tumour resection) + Chemotherapy (anthracycline) + experimental anti-angiogenesis agent	Yes	Yes	No	DOD 13 months later due to no regression of the pelvic tumour
23	Hines	43	Peritoneum (from posterior cul-de-sac through the middle to upper abdomen)	NR	Ca 125: 824 IU/ml	Dysmenorrhea and endometriosis	Surgery (tumour resection) + Hormonal therapy (medroxyprogesterone acetate)	No	Yes	No	FOD 10 months later
24	Murugasu	23	Pouch of Douglas	11	Ca 125: 378 IU/ml; CEA: 13.	Right-sided pelvic pain	Surgery (tumour resection), Chemotherapy (Mesna, adriamycin, ifosfamide), Radiotherapy	Yes	Yes	No	FOD 1 years later
25	Liu	56	Vaginal Vault	16	NR	Urinary incontinence and prolapse in a woman with Vaginal endometriosis (TAH, BSO).	Surgery (tumour resection with adherent structures including rectum and part of the bladder wall) + Chemotherapy (ifosfamide and cisplatin) + Radiotherapy	No	Yes	ERT	FOD
26	Raffaelli	50	Rectovaginal septum	Not reported	NR	Deep dyspareunia, rectal pain, periovulatory pelvic pain in a woman with diagnosis of endometriosis	Surgery (Hysterectomy, left salpingo oophorectomy and partial vaginectomy) + Hormonal therapy (megestrol acetate)	No	Yes	No	Pelvic recurrence 14 months after first surgery (resection of the mass, chemotherapy with ifosfamide and epirubicin-stopped due to intolerance- and radiotherapy 52,90Gy-stopped due to toxic side effect) FOD 9 months after the last surgery
27	Toyoshima	52	Vaginal cuff	11 cm	High level of Ca125		Neoadjuvant therapy and surgical removal of the tumour, the vaginal wall and the greater omentum	Yes	Yes	No	After a month the first lung recurrence treated with chemotherapy, and then a second abdominal recurrence treated with chemotherapy. DOD after 9 months from surgery
28	Kanngurn	48	Pelvic peritoneum	26x26x10 cm	NR	Right lower quadrant pain	Surgery (tumour resection, Hysterectomy, bilateral salpingo oophorectomy) + Chemotherapy (Bleomycin, Etoposide and Cisplatinum × 7 cycles).	Yes	No	No	Abdominal recurrence 8 months later during the fourth cycle of therapy (13, 5 × 7, 8 × 13 cm), lost at follow-up.
29	Chang	37	cul-de-sac	3.5	NR	Vaginal bleeding in a woman with endometriosis (TAH, BSO and hormonal therapy)	Surgery (resection of cul-de-sac tumour, left anterior proctectomy, coloanal anastomosis)	No	yes	yes	FOD 36 months
30	Milam	47	Right inguinal channel	12 × 4	CA 125: 76,8	Persistent and enlarged groin mass	Surgery (tumour resection)	No	Yes	HRT	FOD 12 months later
31	Huang	41	Mesentery of the terminal ileum, right colon and pelvic sidewall	10	NV	Right lower quadrant pain and nausea	Surgery (TAH, bilateral salpingo oophorectomy, omentectomy, resection of cul-de-sac and sigmoid colon nodules, and pelvic and para-aortic lymph node dissection) + Chemotherapy (ifosfamide+ cisplatin)	No	Yes	No	Peritoneal recurrence at 1 month and chemotherapy (liposomal doxorubicin) FOD for 18 months
32	Han	34	Vagina	7 × 6	High value		Tumour resection, hysterectomy and bilateral salpingo-oophorectomy	No	Yes	No	The patient had 4 vaginal recurrences. The first was treated with chemotherapy, the second was treated with surgery, the third with surgery and adjuvant therapy and the fourth with chemotherapy. FOD
33	Maeda	47	Left pelvic side wall	3	NR (LDH level 993 IU/ml)	Acute lower abdominal pain due to pedunculated subserosal myoma	Surgery (tumour resection, bilateral salpingo-oophorectomy, total abdominal hysterectomy)	Yes	Yes	TMX	Four weeks later 8 cm recurrent tumour in the right pelvis treated with salvage surgery. One month after second surgery recurrent tumour in pelvis and upper abdomen that was treated with salvage chemotherapy (liposomal doxorubicin), 5 cycles). FOD three months after chemotherapy.
34	Patrelli	49	Pouch of Douglas	10 × 6	CA 125 and Ca 19–9 slightly elevated	Pelvic pain	Surgery (tumour resection)	Yes	No	No	Eighteen months later recurrence of posterior vaginal fornix (resected), 6 months after recurrence another posterior vaginal fornix recurrence (radical hysterectomy with bilateral salpingo-oophorectomy, and pelvic lymphadenectomy and Radiotherapy-50Gy-) FOD
35	Clarke	50	Pelvic peritoneum (partially adherent to the posterior uterine serosa)	34x14x7	NR	NR	Surgery (omentectomy, appendicectomy, removal of small bowel mesenteric implants)	NR	NR	NR	
36	Karateke	26	Pouch of Douglas	18	NR	Lower abdominal distention and left lower quadrant pain	Surgery (tumour resection)	No	Yes	No	FOD 24 months later
37	Yang	36	Rectum	2,5 × 2	NR	Loose stool, dysmenorrhea, deep dyspareunia, haematochezia	Surgery (tumour resection)	No	Yes	No	FOD 60 months
38	Kar	30	Omentum	NR	NR	Abdominal distension due to abdominal mass and free fluid	Surgery (hysterectomy, bilateral salpingo oophorectomy, omentectomy) and preoperative chemotherapy	Yes	Yes	No	NR
39	Pontrelli	58	Vagina	5 cm	NR	Bleeding vaginal lesion	Vaginectomy and parametrectomy using the laparoscopic approach, total colpectomy and partial cystectomy. After that the patient was candidate for progestin therapy.	Yes	Yes	Yes	FOD at November 2016
40	Mandato	79	Abdominal Peritoneum	16 × 11	NV	Abdominal distension	Surgery (tumour resection, bilateral salpingo-oophorectomy, total hysterectomy) Residual absent	Yes	No	No	4 weeks later had a pelvic recurrence; DOD 4 months after diagnosis

*Abbreviations:* *AWD* alive with disease, *DOD* died of disease, *NER* no evidence of recurrence, *UK* unknown, *NR* not reported, *NV* normal value, *FOD* free of disease, *CHT* chemotherapy, *MTX* methotrexate, *RT* radiotherapy, *Cyt* cyclophosphamide, *MPA* medroxyprogesterone acetate

Generally, uterine AS presents clinically indolent behaviour, whereas AS with sarcomatous overgrowth is extremely aggressive [31] and is characterized by recurrence and metastasis at an early stage [40, 41]. Sarcomatous overgrowth is characterized by the presence of a high-grade sarcomatous component in at least 25% of the tumour [42].

A recent national cancer database study reported survival data from 2205 women with AS arising from the corpus uterus, cervix and ovary, but no consistent data regarding vaginal or extra-genital AS are available because these are extremely rare sites for AS [43]. Uterine AS is the rarest form of uterine sarcomas representing only ∼0.2% of all uterine malignancies. It has an age-adjusted incidence of 2 per 1000,000 for Caucasians, 3 per 1000,000 for African Americans, and 1 per 1000,000 for other ethnic groups in the US population [44, 45].

Extra-genital AS is so rare that it has not been possible to develop clear guidelines regarding treatment and prognosis [35].

Here, we reported a case of primary peritoneal AS with sarcomatous overgrowth but no associated endometriosis and reviewed all cases of AS arising outside of the uterus and outside of the ovaries published since 1974 to identify the best treatment options and clarify outcomes.

## Methods

We report the clinical data, preoperative imaging, pathological findings and follow-up data for a case of primary peritoneal AS with sarcomatous overgrowth. We also performed a systematic review of the literature to collect reports on AS arising outside of the uterus and outside of the ovaries. With the term “uterus” we mean the whole organ without distinction between uterine corpus and cervix. We mean with the term “extra-uterine” all AS arising outside of the uterine corpus or of the cervix.

### Systematic review of the literature

We collected and analysed articles published on AS between January 1974 and October 2016 using PubMed as a database and the following search terms: “peritoneal mullerian adenosarcoma”, “primitive peritoneal mullerian adenosarcoma”, “primary peritoneal mullerian adenosarcoma”, “extra-uterine mullerian adenosarcoma”, “primitive extra-uterine mullerian adenosarcoma”, “primary extra-uterine mullerian adenosarcoma”, “extra-uterine mesodermal adenosarcoma”, “primitive extra-uterine mesodermal adenosarcoma”, “primary extra-uterine mesodermal adenosarcoma”, “primary extra-genital adenosarcoma”, “primitive extra-genital adenosarcoma”, “primary extra-genital mullerian adenosarcoma”, and “primitive extra-genital mullerian adenosarcoma”. After selecting for cases arising outside of the uterus and outside the ovaries, 32 reports of extra-genital AS and 9 of vaginal AS were found and included in this systematic review For each case the following data were extracted and collected in a database: age, tumor size, tumor site, previous diagnosis of endometriosis, sarcomatous overgrowth, heterologous sarcomatous differentiation therapy, presence of recurrences, recurrence site, treatment after recurrence and follow up status and time. All dichotomic parameters were codified as 0 (absent) or 1 (present), while for all cases age was reported in years, follow up was reported in months and tumor size was reported in centimetres. When a patient experienced more than one recurrence all events were reported. Missing data were indicated as not reported (NR) in database.

### Statistical analysis

Statistical analysis was performed using R-3.2.3 software. Associations between clinical and pathological parameters in different subgroups of patients were assessed using linear models and Fisher’s exact test.

Overall survival (OS) was computed as the time period from the date of surgery to either the date of death or last follow-up. Disease-free survival (DFS) was computed as the disease-free period from the date of surgery to the date of relapse or last follow-up. Survival curves were plotted using the Kaplan–Meier method and differences between curves were assessed by Log-Rank test.

Test were considered statistically significant with a P value lower than 0.05.

## Case presentation

**Fig. 1:**
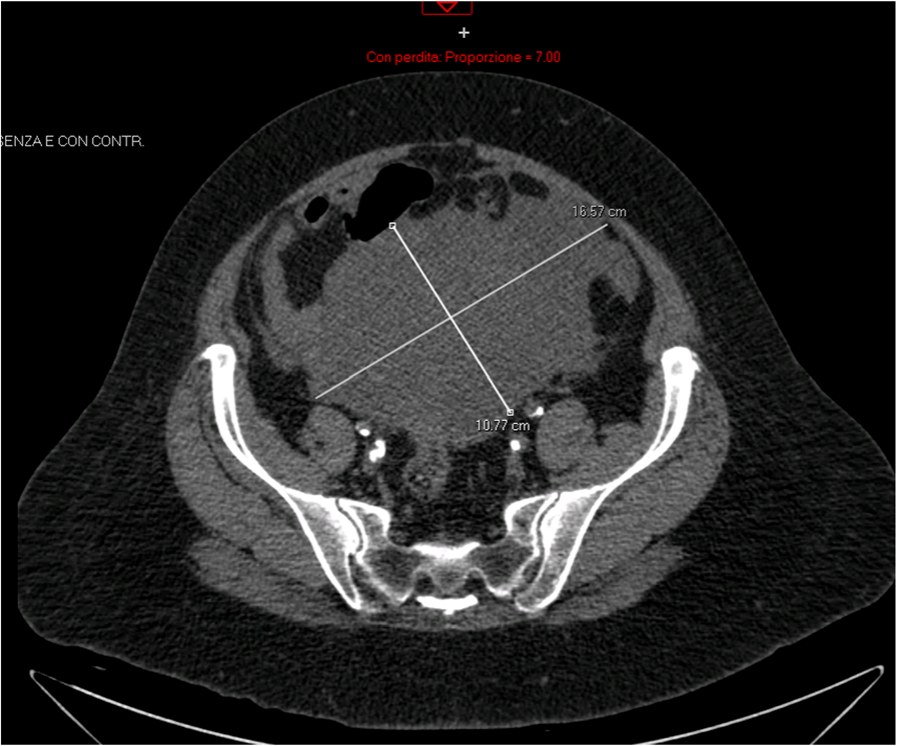
Computed tomography scan showing a mass of 16 × 11 cm

A 79-year-old woman was referred to the Department of Obstetrics and Gynecology with an abdominal mass discovered on a computed tomography scan (CT) performed following right iliac artery angioplasty. The scan revealed a 16 × 11 cm mass resembling a fibroid with a haemorrhage (Fig. 1).

Her history included type 2 diabetes, hypertension, hypercholesterolemia, glaucoma, hypothyroidism, and stage III chronic obstructive arteriopathy of the right leg, and she underwent a left carotid thromboendarterectomy 1 year prior to admission. Her gynaecological history was negative. She complained only of abdominal distension and pressure. A transvaginal and transabdominal ultrasound examination revealed a multicystic mass resembling an ovarian tumour arising from the pelvis and extending up to the abdomen. Three weeks later, a laparotomy was performed, and a peritoneal mass arising from Douglas peritoneum was found and resected. The uterus and adnexa appeared normal, and a supra-cervical hysterectomy with bilateral salpingo-oophorectomy was performed. On frozen sections, the mass was identified as a primary sarcoma of the peritoneum with areas of chondroliposarcoma and rhabdomyosarcoma differenzation. No macroscopic residual disease was present (R0). Final pathology diagnosed a malignant peripheral nerve sheath tumors with divergent differentiation (osteosarcoma, chondrosarcoma, angiosarcoma rhabdomyosarcoma, glandular component), grade 3 according to the French Federation of Cancer Centers Sarcoma Group (FNCLCC) grading system.

**Fig. 2:**
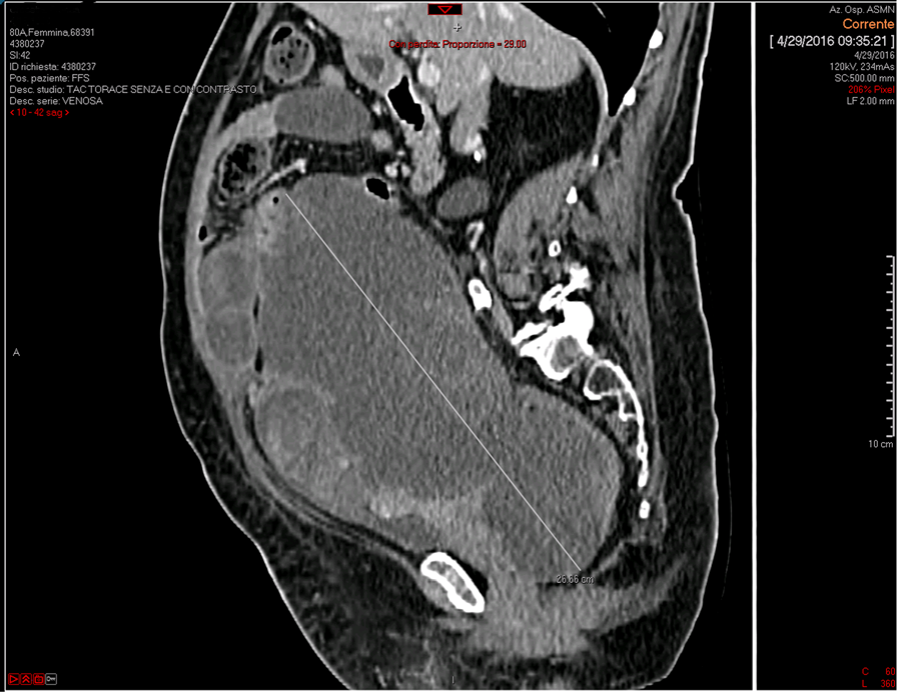
Pre-chemotherapy computed tomography scan taken 4 weeks after surgery revealing a new, multicystic mass (27 × 15 cm)

**Fig. 3:**
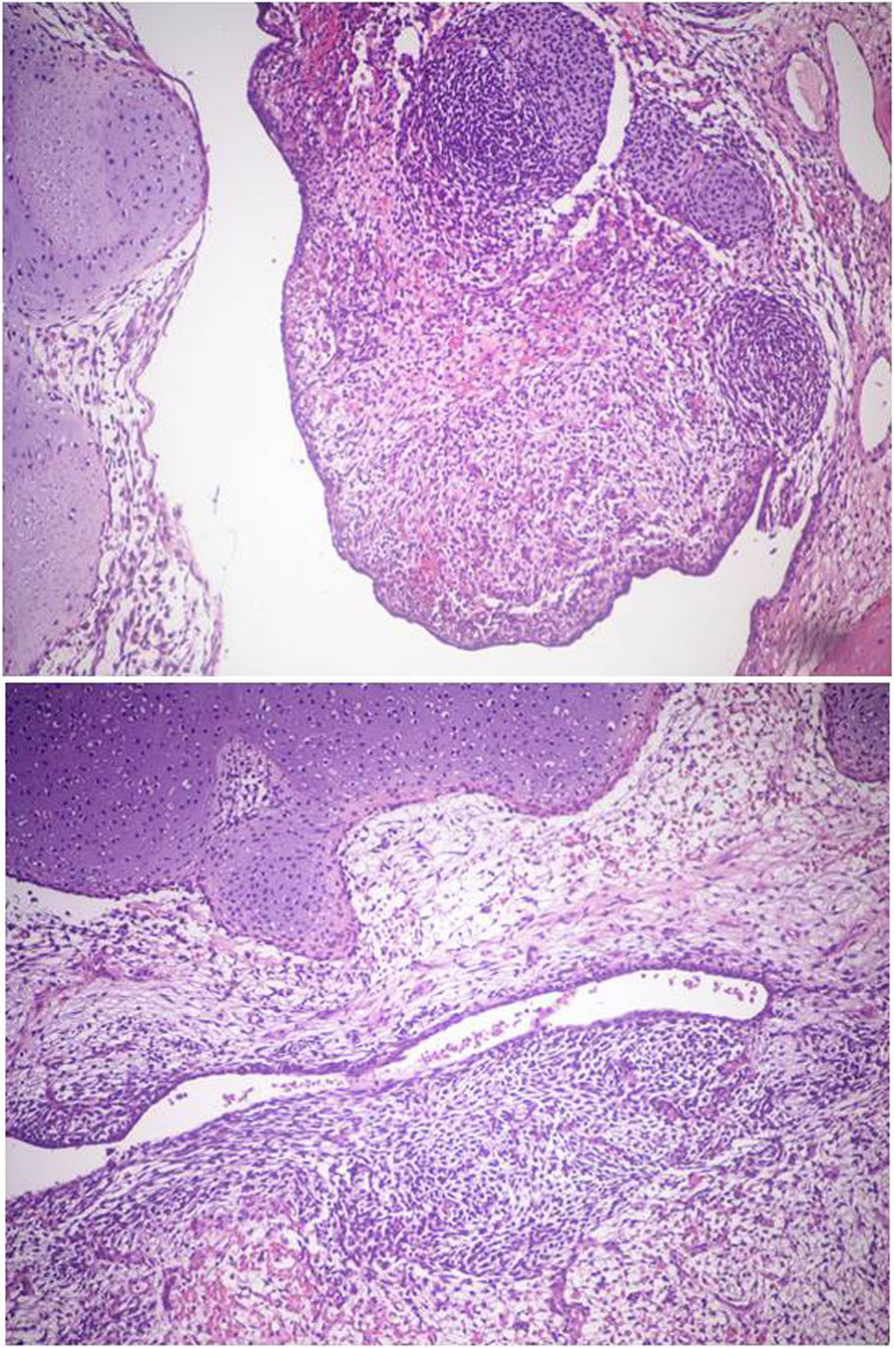
Medium-power view of the neoplasia, showing both the epithelial component and the undifferentiated spindle cell component, admixed with areas of cartilaginous differentiation (haematoxylin-eosin stain, 10X)

**Fig. 4:**
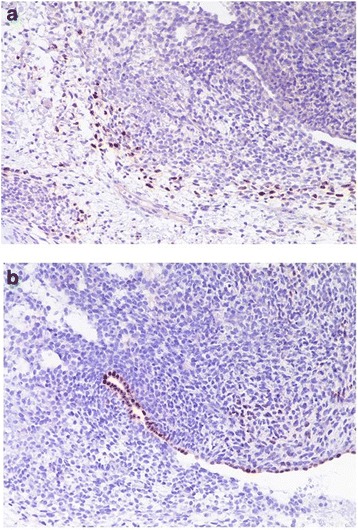
(**a**) Small areas with rhabdomyoblastic differentiation within the spindle cell areas (myogenin immunostain, haematoxylin counterstain, 20X); (**b**) Epithelial clefts within the neoplastic undifferentiated spindle cells highlighted by PAX8 immunohistochemical stain (PAX8 immunostain, haematoxylin counterstain, 20X)

Adjuvant chemotherapy was planned. Four weeks later, a pre-chemotherapy CT scan revealed a new, multicystic mass (27 × 15 cm) (Fig. 2) with impregnation of the wall, strictly adhering to the inferior side of the sigmoid colon and cecal profile and to the superior side of the bladder. The mass protruded into the left inguinal canal by 2 cm. The patient presented with bilateral hydroureteronephrosis, fever due to wound infection, loss of appetite and weakness. Antibiotic therapy, bilateral stents, and support therapy were administered. Due to the progressive poor condition, the patient died 4 months after diagnosis. Histological slides were reviewed by two external independent expert pathologists (A.P. Dei Tos, Chief of Department of Pathology, Treviso Regional Hospital, Treviso, Italy. C.D.M. Fletcher, Chief of Surgical Pathology, Brigham And Women’s Hospital, Boston, USA) and the final diagnosis was of extra-genital AS with sarcomatous overgrowth (Figs. 3 and 4).

## Results

### Clinical features

Table 1 shows the main clinical features of all 41 AS cases reported in literature and of our case.

The 41 affected patients ranged in age from 16 to 83 years (mean, 44.5 years) at presentation, and 2/41 (4.9%) patients were pregnant at the time of diagnosis.

Overall, 12/32 (37.5%) patients presented with an extra-genital AS arising from the pelvic peritoneum, 5/32 (15.6%) presented with an AS arising from the pouch of Douglas, 2/32 (6.3%) presented with an AS arising from the retroperitoneum, 3/32 (9.4%) presented with an AS arising from the broad ligament, 3/32 (9.4%) presented with an AS arising from the colon, 2/32 (6.3%) presented with an AS arising from the small bowel, 1/32 (3.1%) presented with an AS arising from the bladder, 1/32 (3.1%) presented with AS arising from the omentum, 1/32 (3.1%) presented with an AS arising from the inguinal canal, 1/32 (3.1%) presented with an AS arising from the liver, and 1/32 (3.1%) presented with an AS arising from the mesentery of the terminal ileum. Overall, 9/41 (21.9%) patients had an AS localized in the vagina: 7/9 (77.8%) cases were in the vaginal cuff, 1/9 (11.1%) case was in the paracolpium, and 1/9 (11.1%) case was in the recto-vaginal septum.

Information on tumour size was available for 33/41 (80.5%) patients. The sized ranged from 2.5 to 34 cm with a mean size of 12.2 cm (SD +/− 6.0). Tumour weight was reported for 1 case (13 k) [20].

Symptoms were reported for 34/41 (82.9%) patients. Abdominal/pelvic pain was reported for 14/34 (41.2%) patients, urinary disorders for 9/34 (26.5%), anorexia-weight loss for 3/34 (8.8%), abdominal pressure for 3/34 (8.8%), dysmenorrhea for 2/34 (5.9%), bleeding for 4/34 (11.8%), constipation for 1/34 (2.9%), low back pain for 1/34 (2.9%), fatigue for 1/34 (2.9%) and thrombophlebitis for 1/34 (2.9%).

Tumor markers were reported in 13/41 (31.7%) patients, two patients had normal value [20, 31] and 11 patients had elevated value [12, 15, 17, 18, 23, 24, 27, 30, 32–34] (Table 1). Seven of eleven (63.6%) patients had elevated serum levels of CA 125 [12, 15, 18, 23, 27, 30, 32], 2/11 (18.2%) patients had elevated serum levels of both CA 125 and CA 19–9 [17, 34], 1/11 (9.1%) patient had elevated serum levels of both CA125 and CEA [24], 1/11 (9.1%) had elevated serum level of LDH [33].

Overall, 8/41 (19.5%) patients had received hormonal therapy: two patients received hormone replacement therapy (HRT) [17, 30], two patients received oestrogenic replacement therapy [19d,25], one patient received tamoxifen [16], one patient received oestrogen-progestin therapy [39], and in two patients the hormonal therapy was not specified [21, 29].

### Treatment

AS was treated by surgical resection in 38/41 (92.7%) patients: 5/38 (13.2%) patients underwent partial resection, and 33/38 (86.8%) underwent total resection. Of the 38 patients who received surgical treatment, 18 (47.4%) underwent resection of only the tumour [5abc, 7, 8, 9, 11, 13, 18, 19d, 20, 22, 23, 24, 30, 34, 36, 37]; four (10.5%) underwent tumour resection, hysterectomy and bilateral salpingo-oophorectomy [14, 28, 32, 38]; and 16 (42.1%) underwent extensive surgery involving other organs such as the bowel [12, 15, 19a, 19b, 19c, 25, 29, 31, 35] and liver [17].

Moreover, 12/38 (31.69%) patients had received previous total hysterectomy with bilateral salpingo-oophorectomy for benign disease [5A, 5B, 5C, 6, 10,11,13,16,17,22,25,29]; 15/38 (39.5%) patients received total hysterectomy with bilateral salpingo-oophorectomy for AS treatment [8,14,15,19A,19B, 20,22,23,26,28,30–33, 35,38]. Particularly, 13 patients were younger than 40 years at the diagnosis of AS and 4/13 (30.8%) underwent total hysterectomy with bilateral oophorectomy for AS treatment. In 13/41 (31.7%) patients menopausal status was not reported. Moreover, 17/41 (41.5%) [5a–c, 6, 10, 11, 13,16, 17, 19c, 21, 22, 25, 27, 29, 30, 39] patients were in postmenopausal stage, 11/41 (26.8%) patients were at premenopausal stage [4, 8, 9, 12, 14, 18, 24, 31, 36–38] and 4/11 (36.4%) received bilateral salpingo-oophorectomy during AS treatment [8, 14, 31, 38].

Overall, 16/38 (36.6%) surgical patients received additional therapy: 13/38 (34.2%) received adjuvant therapy, and 3/38 (7.9%) received neo-adjuvant therapy [5a, 7, 11, 14, 20, 22,23, 24, 25, 26, 28, 31, 39]. Additionally, 3/38 (7.9%) patients received chemotherapy [22, 28, 31], 2/38 (5.3%) patients received radiotherapy [5a, 11], 3/13 (7.9%) patients received chemo-radiotherapy [7, 24, 25], 4/38 (10.5%) patients received hormonal therapy [20, 23, 26, 39], and 1/38 (2.6%) patient received radiotherapy and hormonal therapy [14]. In total, 2/38 (5.3%) patients were treated with neoadjuvant chemotherapy [14 (methotrexate), 27], and 1/38 (2.6%) patient was treated with neoadjuvant radiotherapy [15].

AS was not treated with surgery in 3/41 (7.3%) patients. In the first patient AS was misdiagnosed with coriocarcinoma and was treated with chemotherapy but at postmortem examination the final diagnosis of retroperitoneal AS was done [4]. The second patient had received a hysterectomy for leiomyoma twenty years before underwent to diagnostic laparoscopy for right pelvic mass. At laparoscopy both ovaries were normal and a biopsy of the mass diagnosed an AS. The second patient was treated with only radiotherapy [6]. The third patient had received a hysterectomy for leiomyoma 4 years before multiple vaginal operations for recurrent vaginal endometriosis were performed [16]. The third patient was treated with chemotherapy and hormonal therapy (tamoxifen) [16].

### Risk factors

A total of 25/41 (61.0%) patients had received a previous diagnosis of endometriosis [5c-10-11-15-16-17-19abc-21-22-23-24-25-26-27-29-30-31-32-33-36-37-38,39] (Table 2).

**Table 2:** Clinical features and follow up data of 41 extra-uterine and extra-ovarian mullerian adenosarcoma according to histological features

	Total population	Endometriosis		*P* value	Overgrowth		*P* value	Heterologous sarcomatous differentiation		*P* value
	*n* = 41 n (%)	No *n* = 16 n (%)	Yes *n* = 25 n (%)		No *n* = 32 n (%)	Yes *n* = 9 n (%)		No *n* = 37 n (%)	Yes *n* = 4 n (%)	
Age (mean ± SD), years	44.5 ± 15.0	43.1 ± 15.8	45.5 ± 12.7	0.597	45.4 ± 13.9	41.4 ± 14.6	0.463	46.4 ± 13.0	27.5 ± 11.8	0.009
Size (mean ± SD), mm	12.2 ± 6.9	13.4 ± 8.3	11.0 ± 5.3	0.319	12.5 ± 6.7	11.0 ± 8.1	0.659	11.9 ± 6.8	14.7 ± 8.5	0.517
Site				0.066			1			1
Extra-genital	32 (78.0)	15 (93.8)	17 (68.0)		25 (78.1)	7 (77.8)		29 (78.4)	3 (75.0)	
Vagina	9 (22.0)	1 (6.2)	8 (32.0)		7 (21.9)	2 (22.2)		8 (21.6)	1 (25.0)	
Endometriosis							1			0.281
No	16 (39.0)	–	–	–	13(40.6)	3 (33.3)		13 (35.1)	3 (75.0)	
Yes	25 (61.0)	–	–	–	19 (59.4)	6 (66.7)		24 (64.9)	1 (25.0)	
Overgrowth				1						1
No	32 (78.0)	13 (81.3)	19(76.0)		–	–		29 (78.4)	3 (75.0)	
Yes	9 (22.0)	3 (18.7)	6 (24.0)		–	–		8 (21.6)	1 (25.0)	
Treatment				0.557			1			0.101
Surgery	22 (53.7)	9 (56.3)	13 (52.0)		18 (56.3)	4 (44.4)		19 (51.4)	3 (75.0)	
Surgery + additional treatments	16 (39.0)	5 (31.2)	11 (44.0)		11 (34.4)	5 (55.6)		16 (43.2)	0 (0.0)	
No Surgery	3 (7.3)	2 (12.5)	1(4.0)		3 (9.4)	0 (0.0)		2 (5.4)	1 (25.0)	
Surgical Approach				0.345			1			0.327
Complete resection	33 (80.5)	11 (68.8)	22 (88.0)		25 (78.1)	8 (88.9)		30 (81.1)	3 (75.0)	
Partial resection	5 (12.2)	3 (18.7)	2 (8.0)		4 (12.5)	1 (11.1)		5 (13.5)	0 (0.0)	
No surgery	3 (7.3)	2 (12.5)	1 (4.0)		3 (9.4)	0 (0.0)		2 (5.4)	1 (25.0)	
Lost in follow up	6 (14.6)	4	2		4	2		6	0	
Status at last follow up (35 patients)				0.002			0.843			0.278
FOD	22 (62.9)	3 (25.0)	19 (82.6)		17 (60.7)	5 (71.4)		19 (61.3)	3 (75.0)	
AWD	4 (11.4)	3 (50.0)	1 (4.3)		4 (14.3)	0 (0.0)		3 (9.7)	1 (25.0)	
DOD	9 (25.7)	6 (25.0)	3 (13.0)		7 (25.0)	2 (28.6)		9 (29.0)	0 (0.0)	
Recurrence	18 (51.4)	9 (56.2)	9 (36.0)	0.184	13 (40.6)	5 (55.5)	0.447	15 (45.5)	3 (75.0)	0.340
More than 1 recurrence	9 (25.7)	4 (28.6)	5 (21.7)	1	5 (17.9)	4 (57.1)	0.294	7 (18.9)	2 (50.0)	1
Death patients OS (mean ± sd)		7.0 ± 5.7	46.3 ± 63.9	0.151	6.0 ± 4.0	16.7 ± 17.8	0.179	20.1 ± 37.8	–	*
Patients with recurrence DFS (mean ± sd)		47.9 ± 63.6	28.0 ± 41.3	0.486	12.2 ± 11.4	11.4 ± 12.6	0.897	11.9 ± 12.1	11.6 ± 10.5	1

AWD alive with disease, DOD died of disease, FOD free of disease

*comparison was not possible, because no patients with heterologous sarcomatous differentiation dead during follow up

Endometriosis treatment was not reported for 18/25 (72%) patients [5c,10,11,15,17,19abc,23,24,26,30,31,33,36,37,38], endometriosis was surgically and hormonally treated in 2/25 (8%) patients [29, 39], it was treated surgically in 3/25 (12%) patients [21, 25, 27], it was treated hormonally (aromatase inhibitor) in 1/25 (4%) patient [22], and it was treated with surgery, hormonal therapy and brachytherapy in 1/25 (4%) patient [16]. Overall, 17/25 (68%) patients with endometriosis had an AS with extra-genital localization [5c-10-11-17-19abc-22-23-24-29-30-31-33-36-37-38], and 8/25 (32%) patients had a vaginally localized tumour [15–16–21-25-26-27-32-39]. Moreover, 8/25 (32%) patients showed elevated levels of tumour markers [15, 17, 23, 24, 27, 30, 32, 33]. A total of 7/25 patients received previous hormonal therapy [17, 21, 25, 29, 30, 33, 39]: 2/7 (28,6%) received HRT [17, 30], 1/7 received (14.3%) ERT [25], 1/7 (14.3%) received TMX [33], 1/7 (14.3%) received oestrogenic-progestinic therapy [39], and 2/7 (28,6%) received unspecified hormonal therapy [21, 29].

Overall, 6/25 (24%) patients with endometriosis showed sarcomatous overgrowth [22, 24, 27, 33, 38, 39]: 13/25 (52%) were only surgically treated [5c-10-17-19abc-21-29-30-32-33-36-37], 10/25 (40%) were treated with surgery and adjuvant therapy [11–15–22-23-24-25-26-31-38-39], 1/25 (4%) was treated with neoadjuvant chemotherapy and surgery [27], and 1/25 (4%) was treated with chemotherapy and hormonal therapy without surgery [16].

Heterologous sarcomatous elements were present in 4/41 (9.7%) patients [9, 12, 16, 18], endometriosis was present in one patient [16,], sarcomatous overgrowth was present in one patient [12], surgery was performed in three cases [9, 12, 18], one case received chemotherapy and tamoxifen [16].

### Follow-up data

Follow-up information was available for 35/41 (85.4%) patients (Table 2); 1/41 (2.4%) patient died from a cause other than AS, and 5/41 (12.2%) were lost to follow-up. At the time of follow-up, 22/35 (62.9%) patients were alive and free of disease (FOD), 9/35 (25.7%) patients had died of disease (DOD), and 4/35 (11.4%) patients were alive with disease (AWD).

In the group of nine DOD patients, 4/9 (44.4%) patients died for relapse [5a,11, 20, 27], 1/9 (11.1%) [7] died for progression of disease, 1/9 (11.1%) patient died for treatment complication (postoperatively massive gastric bleeding) [5b], 1/9 (11.1%) patient died for persistent pelvic tumor [22r] and 2/9 (22.2%) patients died for distant metastasis [4, 6].

Information on follow-up time was available for 34 patients: the mean follow-up was 27 months (range, 1–192). Eighteen patients relapsed [5a, 5c, 8, 9, 11, 12, 13, 14, 16, 20, 21, 26, 27, 28, 31, 32, 33, 34], and their mean DFS was 11.8 months (range, 1–36). Two cases were lost to follow-up after recurrence. For the 9/35 (5.7%) patients who died due to disease [4, 5a, 5b, 6, 7, 11, 20, 22, 27], 8/9 (88.9%) patients had extra-genital AS, and 1/9 (11.1%) had vaginal AS [27]. Additionally, 2/9 (22.2%) patients showed both sarcomatous overgrowth and endometriosis [22, 27]. None of these patients received previous hormonal therapy. Of the patients who died because of AS, 4/9 (44.4%) had experienced a relapse [5a, 11, 20, 27]. The number of relapses ranged from one to five with a mean of two. The most common localization for first relapse was the pelvis, but one patient’s first relapse was in the lung [27]. In total, 2/4 (50%) patients with relapse were surgically treated [11, 20], 1/4 (25%) was not treated [5a], and 1/4 (25%) received only chemotherapy. One patient [11] relapsed additional times at prehepatic/intrahepatic sites and in the heart and received multimodal treatment; another patient [27] had a second abdominal recurrence and was treated with chemotherapy. Of the patients who died from disease, the mean OS was 20.1 months (range, 2–120), and of the subgroup of patients who died after recurrence, the mean DFS was 14 months (range, 1–36). At the time of publication, 4/36 (11.1%) patients were alive with disease [5c, 9, 13, 14]: 3/4 (75%) had an AS with extra-genital localization [5c, 9, 14], and 1/4 (25%) had an AS with vaginal localization [13]. None experienced sarcomatous overgrowth, and 1/4 (25%) had a previous diagnosis of endometriosis [5c]. None had previously received hormonal therapy. All patients alive with disease had at least one relapse. The number of relapses ranged from one to four with a mean of 2.2 (Table 2). Overall, 22/35 (62.9%) patients were FOD at the time of publication. Of these, 14/22 (63.6%) had not experienced relapse [10, 15, 17, 18, 19ab, 23, 24, 25, 29, 30, 36, 37, 39], 11/14 (78.5%) had an AS with extra-genital localization [10, 17, 18, 19ab, 23, 24, 29, 30, 36, 37], 3/14 (21.4%) had an AS with vaginal localization [15, 25, 39], 2/14 (14.3%) had a tumour with sarcomatous overgrowth [24], 13/14 (92.8%) had a previous diagnosis of endometriosis [10, 15, 17, 19ab, 23, 24, 25, 29, 30, 36, 37, 39], 9/14 (64.3%) were only surgically treated [10, 17, 18, 19ab, 29, 30, 36, 37], and 5/14 (35.7%) were treated with surgery and adjuvant therapy [15, 21, 24, 25, 39]. Additionally, 5/14 (35.7%) had previously underwent hormonal therapy [17, 25, 29, 30, 39]. Information on follow-up time was available for 13 patients, and the mean follow-up was 21.9 months (range, 1–60 months). A total of 8/22 (36.4%) patients were alive and FOD despite having one or more relapses during follow-up [12, 16, 20, 26, 31, 32 33, 34]. Their number of relapses ranged from one to four with a mean of 1.8. In 3/8 (37.5%) patients, the first relapse was in the pelvis [12, 26, 33]; in 1/8 (12.5%) patient, it was in the peritoneum [31]; in 3/8 (37.5%) patients, it was in the vagina [16, 32, 34]; and in 1/8 (12.5%) patient, it was in the parametrium [21]. The first relapse was surgically treated in 2/8 (25%) patients [33, 34], it was treated with surgery and adjuvant therapy in 2/8 (25%) patients [16, 26], and it was treated with only chemotherapy [12, 31, 32] or only radiotherapy and brachytherapy [21] in 4/8 (50%) patients. Overall, 4/8 (50%) patients experienced a second relapse [12, 32–34]: 1/4 (25%) patient’s second relapse location was in the pouch of Douglas [12], 1/4 (25%) patient’s relapse was in the pelvic peritoneum [33], and 2/4 (50%) patients’ relapses were in the vagina [32, 34]. In 2/4 (50%) patients, the second recurrence was treated only with chemotherapy [12, 33], whereas in 1/4 (25%) patient, it was treated with surgery and adjuvant therapy, and in 1/4 (25%) patient, it was surgically treated. In total, 2/8 (25%) patients had a third relapse: one was in the pouch of Douglas and was treated with surgery and hormonal therapy [34], and the other was in the vagina and was treated with surgery and adjuvant therapy [32]. Finally, 1/8 (12.5%) patient experienced a fourth vaginal relapse that was treated only with adjuvant therapy [32].

Information on follow-up time was available for six patients. They had a mean DFS of 7.8 months (range, 1–18 months) and a mean total follow-up of 21.7 months (range, 1–57).

Of the FOD patients who experienced at least one recurrence during follow-up, 4/8 (50%) had AS with a peritoneal localization [12, 31, 33, 34] and 4/8 (50%) with a vaginal localization [16, 21, 26, 32], 3/8 (37.5%) showed sarcomatous overgrowth [12, 33, 34], and 6/8 (75%) had a diagnosis of endometriosis [16, 21, 26, 31–33]. In total, 5/8 (62.5%) patients were only surgically treated [12, 21, 32–34], 2/8 (25%) were treated with surgery and adjuvant therapy [26, 31], and 1/8 (12.5%) was treated only with chemotherapy and hormonal therapy [16]. Additionally, 2/8 (25%) had previously received hormonal therapy.

**Fig. 5:**
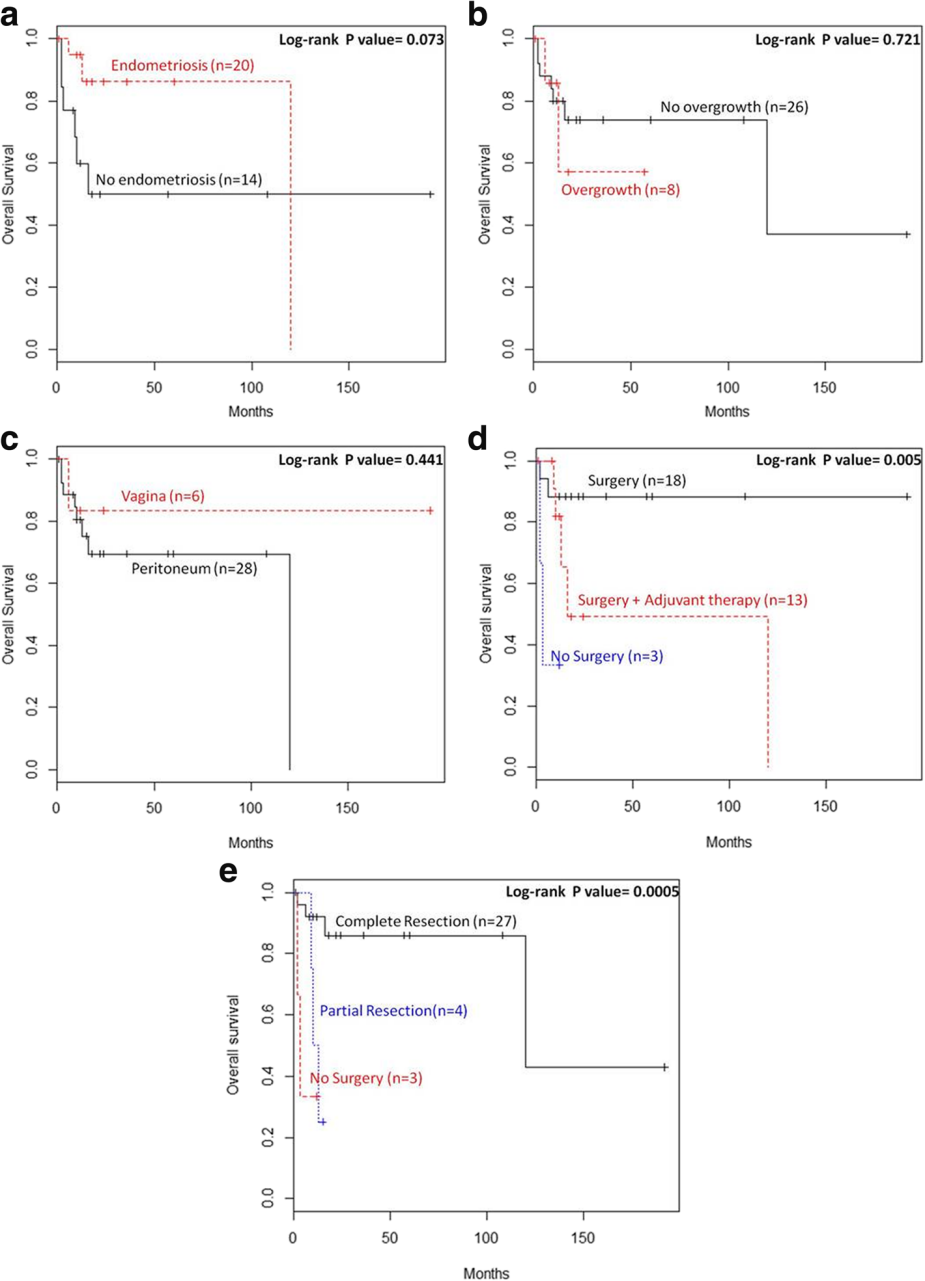
Impact of (**a**) endometriosis, (**b**) sarcomatous overgrowth, (**c**) site of tumor localization, (**d**) treatment, (**e**) surgical approach on OS of patients with extra-uterine AS

**Fig. 6:**
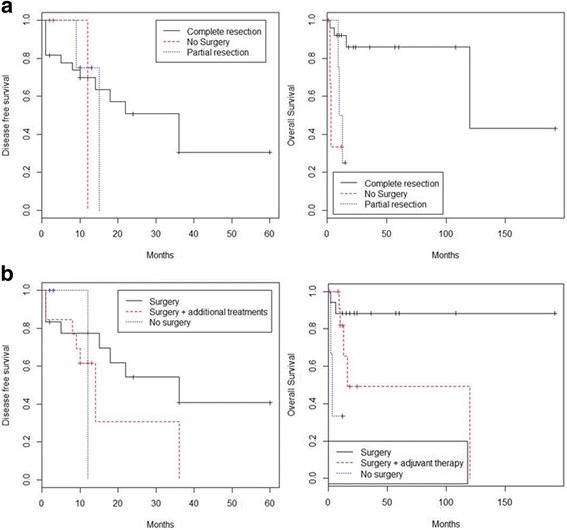
Impact of (**a**) endometriosis, (**b**) sarcomatous overgrowth, (**c**) site of tumor localization, (**d**) treatment, (**e**) surgical approach on DFS of patients with extra-uterine AS

Kaplan Meier curves were used to evaluate the impact of AS pathological characteristics (endometriosis, sarcomatous overgrowth and site of localization) and AS treatment (no surgery, surgery, complete resection, partial resection and adjuvant therapy) on OS (Fig. 5) and DFS (Fig. 6). Statistical comparison of OS Kaplan Meier curves showed a significant difference in survival distribution (log-rank *P* value = 0.005) between patients who received different therapy; in particular patients who received only surgery showed a trend of survival higher than those who received both surgery adjuvant therapy or only adjuvant therapy (Fig. 5d). Moreover, patients with AS treated with complete resection presented better OS than women with partially resected AS or not surgically treated AS (log-rank *P* value = 0.0005) (Fig. 5e).

Evaluation of Kaplan Meier curves referred to DFS showed a significant difference in DFS distribution between patients presenting or not presenting a previous diagnosis of endometriosis (log-rank *P* value = 0.020). Endometriosis resulted to improve DFS of patients with extra-uterine AS (Fig. 6a).

## Discussion

Since 1974, when the first case of an extra-uterine AS was described by Clement and Scully, only 41 cases of extra-uterine or extra-ovarian AS have been reported [3–34] (Table 1).

Here, we reported on 32/41 (78.0%) patients with extra-genital AS and 9/41 (22.0%) patients with vaginal AS. The mean age was 44.5 years (range, 16–83 years; SD +/− 15). The extra-genital AS patients had a mean age of 42.8 years (range, 16–83; + − 14.7), and the vaginal AS patients had a mean age of 50.8 years (range, 42–65 years; SD +/− 9.2). According to previous studies, extra-uterine AS occurs in younger women than uterine AS (median age, 58 years) [1–37].

Unlike prior studies of uterine AS, for which bleeding was the most common presentation symptom [1, 37], in our review, the most common presentation symptom resulted from the large abdominal masses of the AS growths in some patients, with some tumours reaching a size of 34 cm [35] or 13 k [20]. Typically, extra-uterine AS is a large, partly cystic mass with an irregular and lobulated surface [1]. Heterologous elements have been reported in AS from all sites [22], which might cause misdiagnosis of chondroliposarcoma of the peritoneum. It should also be considered that evaluations of frozen sections are less effective when dealing with a huge mass (maximum size: 27 cm). Heterologous elements may portend a poorer prognosis, particularly the rhabdomyoblastic differentiation [46]. Four AS patients included in our review presented heterologous elements [9, 12, 16, 18]. They were younger than other AS patients, although 75% of patients recurred [9, 12, 16], 75% of patients were FOD [12, 16, 18] at last follow-up (Table 2).

CA 125 was reported in 13/41 (31.7%) patients and was found to be greater in 11/13 (84.6%) patients, suggesting an association with peritoneal involvement and sarcomatous overgrowth, as reported by Inoue [15]. In prior studies, CA 125 was reported in 5/10 (40%) patients [12, 24, 27, 33], and it was found at high levels in 4/4 (100%) patients [12, 23, 27, 30]. CA 125 titres have been well correlated with the clinical course of endometriosis associated with extra-uterine AS [30]. In our review, endometriosis was associated with AS in 25/41 (61%) patients, being present in 8/9 (88%) patients with vaginal AS and in 17/33 (51.5%) patients with extra-genital AS.

AS is the second most common gynaecological malignancy in patients with endometriosis after clear cell carcinoma of the ovary [30, 47]. A review of pathologic slides from 1000 cases of surgically proven endometriosis found a 0.3% rate of AS in cases of extra-ovarian endometriosis [30, 47]. In 2000, Zanetta suggested that chronic stimulation from endogenous or exogenous oestrogen may increase the likelihood of endometriosis-associated carcinogenesis [48].

In our review, only 8/41 (19.5%) patients received hormonal therapy [17, 19d, 21, 25, 29, 30, 33,39] such as HRT [17, 30] ERT [19d, 25] or tamoxifen [16]. However, we identified only two patients with AS associated with severe refractory endometriosis who required surgery with hormonal therapy [29] and one patient who required surgery with hormonal therapy and brachytherapy [16]. Nevertheless, old, recurrent and symptomatic endometriosis should be carefully monitored and possibly excised radically [49].

Although endometriosis may be involved in extra-uterine AS tumourigenesis, it is considered a favourable prognostic factor for this tumour type [29, 30]. Patients with AS associated with endometriosis showed increased DFS than AS patients without endometriosis (Fig. 6a).

No endometriosis was found in our patient. In cases of extra-genital AS without endometriosis, the tumour may arise from pluripotent mesothelial and mesenchymal cells in the pelvic cavity [5]. Our patient presented with an extra-genital AS with sarcomatous overgrowth. Sarcomatous overgrowth is characterized by the presence of a high-grade sarcomatous component in at least 25% of the tumour [42] and is associated with poor prognosis for both uterine and extra-uterine AS. In our review, patients with sarcomatous overgrowth showed a worse DFS than patients without overgrowth but log-rank P value between curves did not result completely significant (Fig. 6b).

In a recent retrospective study, patients with uterine AS showed a median OS of 161 months [43]. As reported by Murugasu [24], extra-genital AS is more aggressive than uterine AS, with AS recurring in 60% of extra-genital AS patients compared with 23% of uterine AS patients. In our review, 12/28 (42.9%) patients with extra-genital AS relapsed. Haematogenous metastases have been found in 33% of extra-genital AS patients compared to 2% of uterine AS patients. Death due to tumour occurred in 40% of extra-genital AS patients compared to 10% of uterine AS patients [24]. In our review, 8/28 (28.6%) patients with extra-genital AS died of disease. The aggressiveness of extra-genital AS may be due to failure of the uterine myometrial wall as a barrier. Extra-genital AS is typically quite large at presentation and can easily spread to the peritoneum, to abdominal and pelvic organs, and to blood vessels. For this reason, it can easily cause bowel obstruction, and complete cytoreduction is not always easily achieved.

Surgical treatment, particularly complete surgical resection, represents the best course of action for AS. Patients with extra-uterine AS who received only surgery remained free of disease and never relapsed after treatment. Patients who underwent complete resection showed a better OS distribution than patients who underwent partial resection (Fig. 5e). Endometriosis, sarcomatous overgrowth, tumour size and age were not correlated with resection type. Because the number of patients who were not surgically treated was small, these results require further confirmation.

Moreover, survival seems to be not improved by bilateral salpingooophorectomy. Ovarian preservation for uterine or cervical AS may be feasible among premenopausal women. Indeed, women who underwent hysterectomy with salpingooophorectomy for uterine AS did not have longer survival than women who underwent only hysterectomy [43]. This finding was not tested in our review because we had a lot of missing data about postmenopausal status.

The OS of AS patients who received only surgery resulted more favourable than that of patients who received surgery with additional treatment or who did not undergo surgery (Fig. 6b). Probably, patients submitted to an exclusive surgery presented a completely resectable disease thanks to the biology of tumor or to the skills of surgeon. However, there were no differences in DFS between these three groups (Fig. 6b).

Different adjuvant treatments were delivered. Three patients received chemotherapy alone (anthracycline with an anti-angiogenesis agent [22]; bleomycin, etoposide and cisplatinum [28]; ifosfamide with cisplatin [31]), 3 received chemotherapy associated with radiotherapy (cytoxan + 4000 rad of radiotherapy [7]; mesna, adriamycin, ifosfamide, carboplatinum and pelvic radiotherapy [24]; ifosfamide and cisplatin plus pelvic radiotherapy [25]), and two received only radiotherapy [5a-11].

Unfortunately, our review was limited by the low number of AS cases, by the lack of data and by short follow-up time reported in literature therefore statistical analysis was limited to Kaplan Meier curves comparison. Furthermore, our AS case was characterized by an extremely unusual aggressive clinical course that it seems to be not representative of AS common biological behavior. However, some indications may be suggested.

## Conclusion

In summary, extra-uterine AS, particularly cases arising from extra-genital regions, is an extremely rare tumour. They are typically found in younger women than are uterine AS, and they usually involve huge, polylobate masses that can easily spread into surrounding organs and blood vessels. For extra-uterine AS, endometriosis represents a positive prognostic factor and sarcomatous overgrowth a negative prognostic factor; we could not asses the prognostic effect of heterologous sarcomatous elements for the scant number of cases included; however, independently of sarcomatous overgrowth, extra-uterine AS has a very poor prognosis. Although complete resection is not always feasible, surgery remains the mainstay treatment choice, whereas adjuvant therapy does not appear to be effective in prolonging OS. Surgical treatment of extra-uterine AS often requires an extensive surgery given the possible involving of multiple organs. Moreover, AS patients can be pluri-operated because of previous endometriosis treatment with consequent additional difficulties during surgery. Therefore, since surgery is the only treatment to have an impact on survival, patients should be centralized in qualified surgical oncological centres and operated by experienced surgeons to reduce morbidity and to achieve radical treatment. Nevertheless, centralization might allow the recovery of clinical data and histological samples allowing a revision and a definitive diagnosis. Considering that in the last forty years less than forty cases have been reported in the literature, a worldwide registry is urgently needed to collect data regarding these rare AS to standardize treatment and obtain reliable data on prognosis.

## Abbreviations

AS: Adenosarcoma
AWD: Alive with disease
CI: Confidence interval
CT: Computed tomography scan
DFS: Disease-free survival
DOD: Died of disease
FOD: Free of disease
HR: Hazard ratio
HRT: Hormone replacement therapy
OS: Overall survival
SD: Standard deviation
